# Effectiveness of five personal shark-bite deterrents for surfers

**DOI:** 10.7717/peerj.5554

**Published:** 2018-08-31

**Authors:** Charlie Huveneers, Sasha Whitmarsh, Madeline Thiele, Lauren Meyer, Andrew Fox, Corey J.A. Bradshaw

**Affiliations:** 1 Southern Shark Ecology Group, College of Science and Engineering, Flinders University, Adelaide, Australia; 2 Fox Shark Research Foundation, Adelaide, South Australia, Australia; 3 Global Ecology, College of Science and Engineering, Flinders University, Adelaide, Australia

**Keywords:** Shark repellents, Shark attack, Human-wildlife conflicts, Human protection, Mitigation measures

## Abstract

The number of shark-human interactions and shark bites per capita has been increasing since the 1980s, leading to a rise in measures developed to mitigate the risk of shark bites. Yet many of the products commercially available for personal protection have not been scientifically tested, potentially providing an exaggerated sense of security to the people using them. We tested five personal shark deterrents developed for surfers (*Shark Shield Pty Ltd* [*Ocean Guardian*]* Freedom+ Surf, Rpela, SharkBanz bracelet, SharkBanz surf leash,* and *Chillax Wax*) by comparing the percentage of baits taken, distance to the bait, number of passes, and whether a shark reaction could be observed. We did a total of 297 successful trials at the Neptune Islands Group Marine Park in South Australia, during which 44 different white sharks (*Carcharodon carcharias*) interacted with the bait, making a total of 1413 passes. The effectiveness of the deterrents was variable, with the *Freedom+ Surf* affecting shark behaviour the most and reducing the percentage of bait taken from 96% (relative to the control board) to 40%. The mean distance of sharks to the board increased from 1.6 ± 0.1 m (control board) to 2.6 ± 0.1 m when the *Freedom Surf+* was active. The other deterrents had limited or no measureable effect on white shark behavour. Based on our power analyses, the smallest effect size that could be reliably detected was ∼15%, which for the first time provides information about the effect size that a deterrent study like ours can reliably detect. Our study shows that deterrents based on similar principles—overwhelming a shark’s electroreceptors (the ampullae of Lorenzini) with electrical pulses—differ in their efficacy, reinforcing the need to test each product independently. Our results will allow private and government agencies and the public to make informed decisions about the use and suitability of these five products.

## Introduction

Although shark-human interactions remain rare and unlikely events, their frequency has been increasing globally since the 1980s (Chapman & McPhee, 2016; McPhee, 2014). Growth in human population, habitat modification and destruction, declining water quality, climate change and anomalous weather patterns, and the distribution and abundance of prey have all been proposed to explain this recent increase in the incidence of shark bites and shark bites per capita (Afonso, Niella & Hazin, 2017; Chapman & McPhee, 2016; Lemahieu et al., 2017; McPhee, 2012; Meyer et al., 2018). However, the infrequent occurrence of such events impedes our ability to assess the relative importance of causal factors that might have contributed to the rise in the global and regional number of shark bites (but see Afonso, Niella & Hazin, 2017; Meyer et al., 2018). While the probability of being bitten by a shark is low, and most shark bites result in minor injuries (West, 2011; Woolgar et al., 2001), public perception of the risk of shark bites and ensuing fatality is much higher than reality (Crossley et al., 2014; Myrick & Evans, 2014). The frequent negative framing by the media and user-driven content sites (e.g., YouTube) might have contributed to exaggerating public anxiety about the pervasive presence of sharks and risk that they pose to humans (Muter et al., 2013; Sabatier & Huveneers, 2018). Such heightened public concern has pressured managers and governments to develop and implement new measures that reduce the risk of sharks bites, and provide information to the public to make more informed decisions about using specific areas at particular times.

Prevention and responses to shark bites have varied temporally and regionally, and have included shark hunts, organised shark culling, beach meshing and drumlines, beach closures, shark fences, land- and aerial-based shark spotting, and acoustic telemetry (for a review see Curtis et al. (2012)). While these measures aim to reduce the probability of sharks and humans encountering each other, other measures aim to repel sharks directly from approaching people in the water. These deterrents have been developed to elicit a response by impacting one or more of the shark’s senses, including vision, smell, and electro-reception (see Hart & Collin, 2015). For example, various aposematic colour configurations (i.e., use of colours as anti-predator tactics) have been alleged to repel sharks. Using chemicals as shark repellents has also been proposed (Baldridge, 1990; Rasmussen & Schmidt, 1992; Sisneros & Nelson, 2001). However, the sensitivity of the electro-receptive organ of sharks to strong electric fields and its potential ability to deter sharks have been studied the most (e.g., Huveneers et al., 2013b; O’Connell et al., 2014b).

The rise in shark-human interactions has also led to the emergence of many new personal shark deterrents. The rapid commercial availability of these deterrents has preceded rigorous and peer-reviewed studies to test the effectiveness of these devices, meaning that manufacturers are making claims about their products without rigorous scientific evidence to back them up. If deterrents were not as effective as advertised, it could potentially give users a false sense of security, leading some people to put themselves at greater risk of shark interactions than they normally would because of their reliance on these devices. For example, some surfers and spearfishers might ignore other mitigation measures, such as beach closures, because they feel safe when wearing these products. Whether they are or not is what we aim to demonstrate in this study.

Surfing has been suggested as an activity that exposes people to sharks more than others, because many ideal surfing locations are regions that overlap with the habitats of potentially dangerous sharks, the amount of time surfers spend in the water relative to most other bathers, surfers’ distance from shore, isolation, possible resemblance to natural prey of white sharks (i.e., fur seals, sea lions), and potentially enticing arm and leg movements (Burgess et al., 2010). For example, most bites (63%) in Volusia County, USA between 1982 and 2013 occurred during surfing activities, while surfing has also been implicated in 53% of shark bites in Brazil since 1992 (Chapman & McPhee, 2016) and two-thirds of shark bites in Reunion Island (McPhee, 2014). This has resulted in recent development of personal deterrents to decrease the risk of shark bites to surfers.

Our aims were to test the effectiveness of surfing-specific personal shark deterrents and quantify the behavioural response of sharks exposed to these deterrents. We tested the effects of five deterrents (two electric, two magnetic, and one olfactory-based) on the behaviour of white sharks (*Carcharodon carcharias*) and determined if these deterrents reduce the likelihood of white sharks consuming a bait. Specifically, we assessed and compared the effects of each deterrent on (1) the number of passes to a bait, (2) the minimum distance between a bait and the sharks, (3) the percentage of bait taken, and (4) whether shark behaviour changed with increased exposure to the deterrent (e.g., whether sharks became habituated to the deterrent). We also assessed the effect size that the trials could detect statistically (power analysis) to gauge the experiment’s ability to identify small behavioural changes if present.

## Methods

### Study species and site

In Australia, the white shark is responsible for the most unprovoked bites (19.5% of all shark bites) and the most fatalities (34%) (West, 2011). Our study focused on white sharks because interactions with this species are considered to be a worst-case scenario during which the deterrent is subjected to the most dangerous species. We did all deterrent-testing trials at the Neptune Islands Group Marine Park (35°149S, 136°049E), ∼30 km off the southern coast of South Australia. The area supports the largest colony of fur seals (*Arctocephalus forsteri*) in Australia (Goldsworthy, Shaughnessy & Page, 2014; Shaughnessy, Dennis & Seager, 2005), and is considered an aggregation site for white sharks (Huveneers & Lloyd, 2017). Commercial shark-cage diving has taken place here since the late 1970s and is the only location where such activity is permitted in Australia (Huveneers et al., 2017). We chose this area because of the high likelihood of shark interactions. We did the deterrent testing over 18 days and five separate trips between September 2017 and January 2018.

### Deterrent set-up

**Figure 1:**
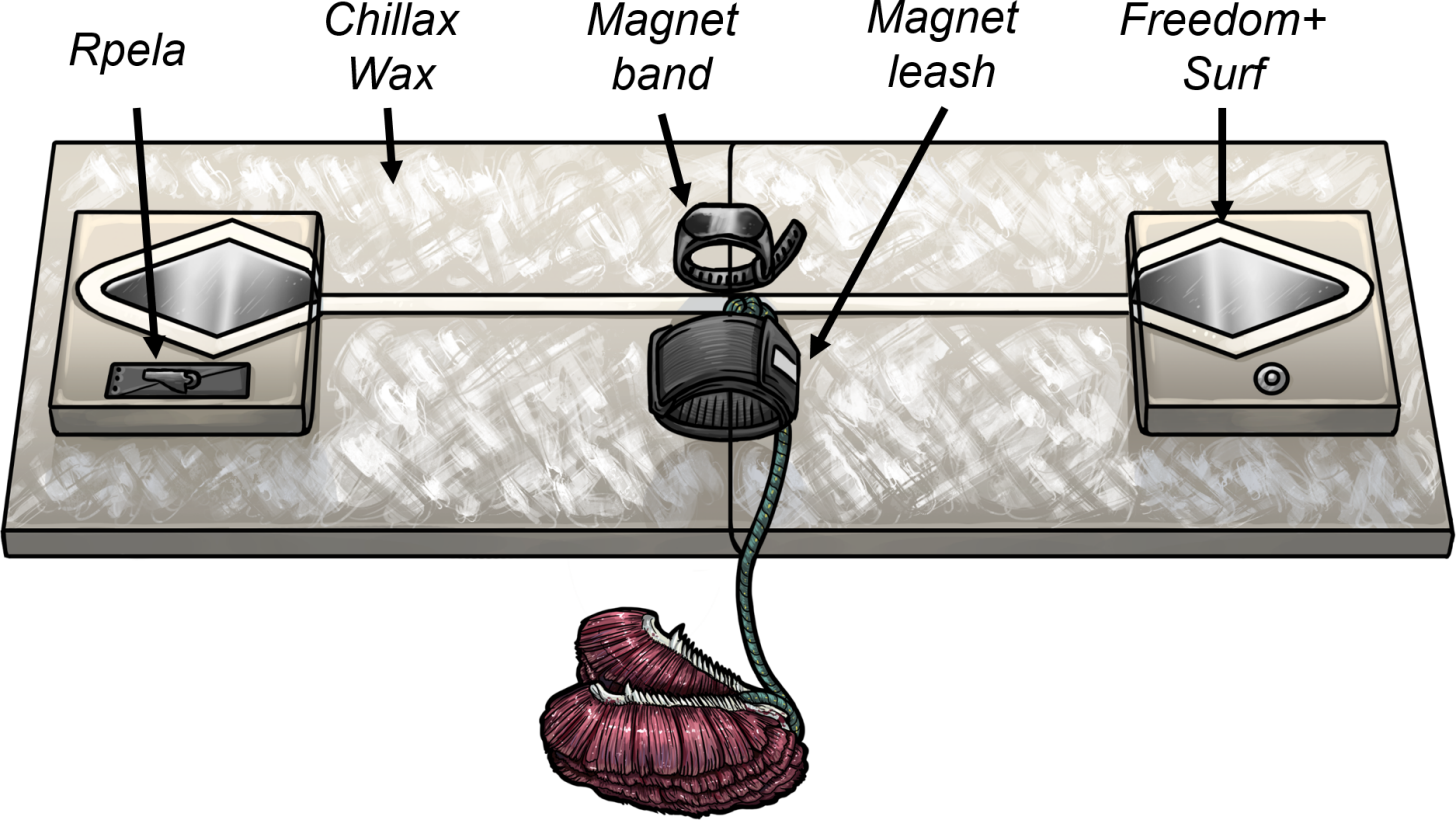
Board set-up used during trials. Illustration of the board set-up (120 × 30 cm) with the five deterrents tested (illustration by René Campbell, Flinders University).

We tested five commercially available deterrents (*Shark Shield Pty Ltd* (*Ocean Guardian*) *Freedom+ Surf, Rpela, SharkBanz bracelet, SharkBanz surf leash* (*Modom*)*,* and *Chillax Wax*; Table 1) using custom-built surfboard replicas (hereafter referred to as ‘boards’). In the case of the *Rpela*, we did the trials first using the commercially available device. We made minor modifications to the electrode size through the trials at the manufacturer’s request, but this did not affect shark responses (see Results). Boards were 120 × 30 cm and made of polystyrene foam covered with layers of fibreglass cloth and epoxy resin, but were strengthened with wood on the sides where the bait was attached. We used six boards, with each having one active deterrent and four replica or dummy deterrents to act as a control (Fig. 1). One board had no active deterrents (i.e., it had the five dummy deterrents) and was used as the control. For example, the *Shark Shield* (*Ocean Guardian*) *Freedom*+ *Surf* (hereafter referred to as *Surf*+) board consisted of an active *Surf*+, regular wax, a replica *Rpela*, and a dummy *SharkBanz bracelet* and *leash*. This experimental set-up allowed us to test for each active deterrent type using a single control board.

**Table 1:** Characteristics of the five commercially available deterrents tested.

**Deterrent type**	**Brand/product**	**Website**	**Abbreviation**	**Characteristics**
olfactory	*Chillax Wax*	http://www.facebook.com/commonsensesurf	*Wax*	ingredients: eucalyptus, chilli, cloves, cayenne pepper, neem, tea tree oil, citronella, coconut, and beeswax
magnetic	*SharkBanz - bracelet*	http://www.sharkbanz.com.au/products/sharkbanz-2	*Magnet band*	grade C8 barium ferrite; BaFe_2_O_4_
magnetic	*SharkBanz - leash*	http://www.sharkbanz.com.au/products/modom-shark-leash	*Magnet leash*	grade C8 barium ferrite; BaFe_2_O_4_
electric	*Ocean Guardian Freedom+ Surf*	https://sharkshield.com/shop/freedom-surf-bundle/	*Surf*+	type: alternative current; voltage: 115 V; frequency: 1.6 Hz; pulse duration: 1.5 ms
electric	*Rpela*	http://www.rpela.com	*Rpela*	type: direct current; voltage: 200 V; frequency: 14.5 Hz; pulse duration: 0.2 ms

### Field sampling

This project was done under the Department of Environment, Water and Natural Resources permit M26609 and Flinders University Animal Ethics Committee approval E446. Across the five sampling trips, we tested each deterrent a total of 50 times within a series of trials. A series of trials consisted of testing each of the five deterrents and the control board (total six trials) in a randomised sequence. This led to a total of 300 trials (50 trials for each of the five deterrents and control).

We attracted white sharks by disbursing into the water a mix of fish oil and minced southern bluefin tuna *Thunnus maccoyii* (i.e., berley or chum) and by deploying sections of tuna attached to a float ∼15 m from the stern of the vessel. Trials commenced after a white shark was sighted near the vessel at least twice within five minutes or when a shark showed consistent interest in the tuna section. We removed and replaced the tuna section with the deterrent set-up, which we deployed when the shark had left the proximity of the vessel. Each trial consisted of the deployment of a tuna gill (∼2 kg), referred to as the ‘bait’, which we attached ∼30 cm beneath the board to replicate the typical distance between a surfer’s foot and the board when surfers sit on their board to wait for a wave. The deterrent set-up was between 5 and 15 m from the vessel and varied depending on wind, swell, tide, and glare conditions, to ensure that observers could identify sharks and record behaviour. We ran trials for 15 minutes or until a shark touched the bait or board with an intent to consume the bait. We repeated trials during which a shark did not approach the board with an intent to take the bait to ensure that the results were not biased by trials during which sharks did not attempt to consume the bait.

During each trial, we deployed a stereo-video unit from the stern of the boat ∼50 cm below the surface to film, measure distance, and enable coding of each trial. The stereo-video unit consisted of two GoPro Hero4 Silver edition cameras mounted in secure, custom-built housings (SeaGIS Pty Ltd, Victoria, Australia) angled 8 degrees inward and set 76 cm distance apart along a metal bar. We calibrated the cameras using EventMeasure (http://www.seagis.com.au/event.html) to take accurate length measurements from the video footage. This software uses 3-dimensional calibration information to calculate distance. Water visibility at the Neptune Islands is typically >10 m, beyond the distances from which the products tested are expected to affect shark behaviour; we therefore contend that visibility did not influence our data.

### Video processing and filtering

We processed and analysed the video footage using EventMeasure. The coders were ‘blind’ because they did not participate in all of the trials and had no prior knowledge of which deterrent was being used when coding videos. We used the following terminology to describe and code shark behaviour following Huveneers et al. (2013b):

*Pass*: a directed swim towards the experimental set-up (each time a shark veered away from the board and swam back we classified it as a new pass);

*Shark identity*: We identified white sharks based on markings on five areas: caudal fin, pelvic fins, first dorsal fin, gills, and pectoral fins using standard methods (Nasby-Lucas & Domeier, 2012; Nazimi et al., 2018). Pigmentation patterns (countershading, rosettes, islets, freckles, spots), notches or scoops, amputations, scoliosis, and scars were all used to recognise individuals. We used videos of sharks turning and showing both sides and side-by-side comparisons of fin silhouettes to link left and right sides of sharks;

*Distance to bait*: distance from the sharks’ nose, where the shark’s sensory organs targeted by the deterrents (ampullae of Lorenzini, nostrils) are located, to the top of the bait;

*Level of intent*: this represented the shark’s motivation when approaching the bait; we categorised this as *high, medium*, or *low* using a combination of factors including shark swimming direction in relation to the bait, swimming speed, and acceleration. *low* = shark moving slowly, not approaching in the direction of the bait and without acceleration; *medium* = shark slowly moving towards the bait without acceleration; *high* = shark approaching the bait at speed or accelerating;

*Approach type*: we categorised the position of the shark in the water column when approaching the bait as either *surface* (horizontal swimming near the surface), *deep* (horizontal swimming >2 m depth), or *vertical* (shark comes up from below the bait and swims vertically in a typical breach approach);

*Reaction*: a behavioural reaction from an individual shark towards the experimental set-up (e.g., tail flick, muscle spasm, head shake, fast direction change).

We determined sex based on clasper presence and measured total body length using EventMeasure. We did not include the effect of shark sex or size in the analysis because potential differences here were beyond the scope of our study. Instead, we included individual shark (shark ID) as a random effect in the models (see below) to account statistically for individual shark behaviour that was independent of deterrent effects (i.e., some sharks might be more inclined to approach closer or more frequently than others). Prior to analysis, we removed passes that had low intent or that were deep and not directed at the board to avoid including behaviours where sharks were not attempting to consume the bait.

### Analysis

The data we collected had two potential analytical biases: temporal correlation and pseudo-replication when the same shark interacted with the deterrent set-up several times within and across trials. For example, sharks might become habituated to the deterrent through repeated exposure. Sharks that consumed the bait might also become less likely to respond to the deterrent because of the positive reinforcement provided by the bait. We investigated whether the effectiveness of the deterrents changed throughout the study (e.g., sharks becoming habituated to the deterrents) by including ‘trial’ as a fixed integer covariate in the models (i.e., ignoring the real elapsed time between successive exposures, but including the information indicating relative serial time; e.g., 2 followed 1) and by plotting the mean distance between the shark and the board, and the number of passes across trials for sharks that interacted with the board for 15 trials or more.

We minimised potential pseudo-replication by testing the effects of deterrents on all response variables using a generalised linear mixed-effects model (GLMM) with individual shark coded as a random effect, and the deterrent used as the fixed effect. We also included trial as fixed-integer effect and the interaction between trial and deterrent to account for potential temporal effects. We included individual shark as a random effect to account for the potential lack of independence in behaviour within individual sharks. We excluded those passes for which we could not identify the shark from this analysis (117 out of 1,413 passes; 8.2%). We determined the most appropriate statistical family and error distribution for each analysis by examining the distribution of the response variable and visually inspecting the residuals for the saturated models. We ran all models for all possible combinations of factors, and compared their relative probability using Akaike’s information criterion corrected for small sample size (AIC_*c*_) (Burnham & Anderson, 2002). The bias-corrected relative weight of evidence for each model, given the data and the suite of candidate models considered, was the AIC_*c*_ weight; the smaller the weight, the lower the probability the model was ‘true’ (Burnham & Anderson, 2002). We also calculated the marginal R^2^ of each resampled GLMM (*R*_m_) as a measure of goodness of fit and the contribution of the fixed effects to explaining variance in the response variable (Nakagawa & Schielzeth, 2013). We also compared the proportion of time the board or baits were touched or taken by sharks between deterrents using the ‘minlike’ method two-sided Poisson exact test from the *exactci* R package (Fay, 2010). We used this test because it is generally more powerful than the central two-sided method (Fay, 2010).

Sharks were likely to respond to the odour from the bait and approach it at different times through the trial. For example, sharks could be within the vicinity of the testing equipment prior to being sighted. We were therefore unable to estimate accurately the amount of time individual sharks took to consume the bait, acknowledging that this variable might be influenced by our inability to detect arrival to the study area. We therefore did not quantify or analyse whether deterrents affected how long it took for the bait to be consumed.

### Power analysis

Using the variance structure of the observed datasets, we determined how much of an increase in the response was required to give statistical evidence for an effect of each deterrent. We first separated each deterrent-control combination for each of three responses: proportion of bait taken, distance to bait, and number of passes. For each subset, we increased the response variable measured for the deterrent trials by 5% increments up to a maximum of 150% of the observed values (i.e., multiplying by a scalar of 1, 1.05, 1.10, …, 1.5). Within each incremented set, we randomly resampled the entire subset with replacement (i.e., all rows, including both control and treatment [deterrent type]) up to the same number of samples, repeating this procedure 1,000 times. For each iteration, we recorded the information-theoretic evidence ratio of the model including only the deterrent fixed effect (i.e., model 3; response ∼ deterrent + shark ID random effect) relative to the intercept-only model (i.e., *w*AIC_*c*_ of model 3 ÷*w*AIC_*c*_ of the intercept-only model). We then plotted the median (and 95% confidence bounds) of model 3 evidence ratio against the response increment. We concluded that an effect could become statistically distinguishable from the control when the most common top-ranked model started to include a *deterrent* effect, and when the evidence ratio became ≫2 (i.e., the *deterrent* model was at least twice as likely to be the true model relative to the intercept-only [no effect]).

## Results

Across the five trips, we did a total of 342 trials, from which we removed 42 from further analysis because no sharks approached the board with an intent to take the bait during these trials. We also removed two trials with the *Surf*+ and one trial with the *Rpela* due to technological issues with the device at the time of deployment. Out of the remaining 297 trials, we recorded 1413 passes from a total of 44 individual sharks. The mean (±  standard error) distance between sharks and baits was 2.30 ± 0.04 m (range: 0–10.8 m; *n* = 1217 passes), which we measured with a precision of 46.5 ± 1.0 mm (2.3–325.4 mm). Passes for which we could not measure distance (*n* = 196) were due to objects (e.g., bubbles from weather conditions, other fish species such as silver trevally) obstructing the field of view or to the framing of the video cutting off the board and bait or the shark. Individual sharks interacted with the board for an average of 29.5 ± 5.3 passes (range: 1–152) and 8.9 ± 1.4 trials (range: 1–42). The mean number of sharks that approached the bait during a trial was 1.3 ± 0.03 (range 1–4). Most (68%) passes occurred at the surface, with only a few (4%) vertical passes. Overall, the distribution of pass type was approximately evenly distributed among the intent classes: 38, 40, and 23% high, medium, and low intent, respectively. However, this distribution reversed between surface and deep passes because most surface passes had high intent and most deep passes had low intent (Fig. S1).

**Figure 2:**
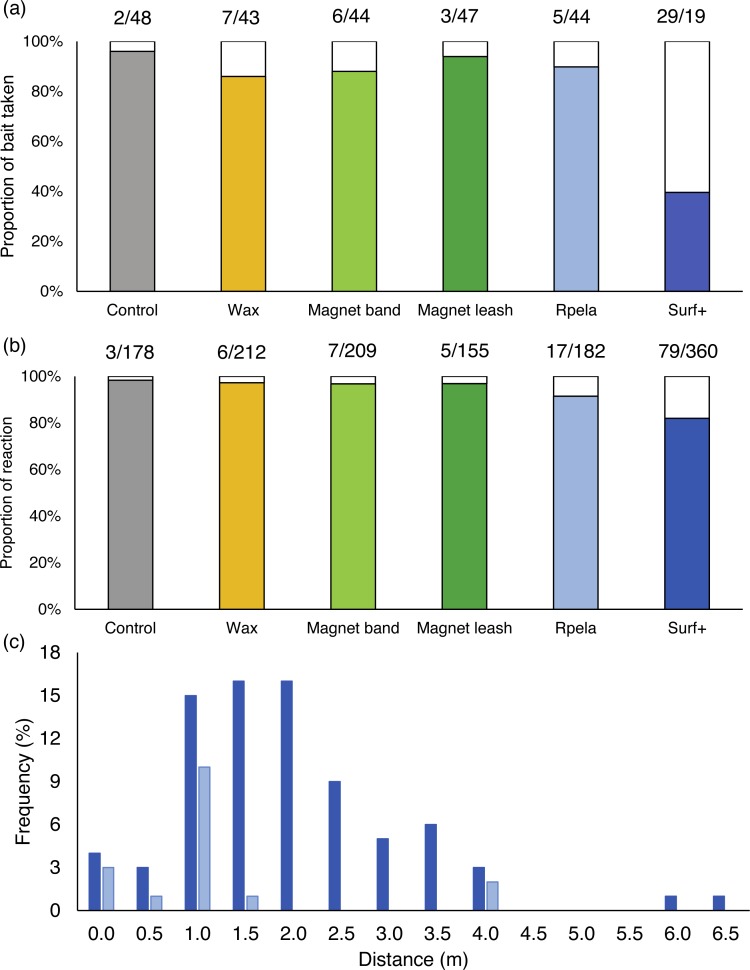
Effects of shark deterrents on white shark behaviour. (A) Percentage of board or bait touched or taken and (B) reaction by white sharks during 15-minute trials with a control board (grey) or one of five deterrents (coloured bar). White bars represent trials when the board and bait were not touched or taken, or without any reaction. Numbers above bars represent (A) the number of trials with board and bait touched/taken or not touched/taken and (B) the number of pass with or without reaction. (C) Frequency distribution of the distance at which white sharks reacted to the *Rpela* (light blue; *n* = 17) and the *Shark Shield (Ocean Guardian) Freedom+ Surf* (dark blue; *n* = 79). Only deterrents for which sharks reacted >15 times are shown.

When no deterrents were active (i.e., on the control board), sharks touched or bit the board or bait (hereafter referred to as an ‘interaction’) prior to the end of the trials 96% of the time. The deterrents had various effects, with the percentage of times sharks interacted with the bait ranging from 94% (*magnet leash*) to 40% (*Surf*+) (Fig. 2A). The *Surf*+ was the only deterrent that had statistical evidence for reducing the percentage of times sharks interacted with the bait (Poisson exact test: *p* < 0.001; *p* > 0.67 for all other deterrents). The *w*AIC_*c*_ top-ranked model (*w*AIC_*c*_ = 0.73) included deterrent, with the deterrent factor explaining ∼23% of the variance (Table 2A). The *Surf*+ had the largest effect on the percentage of time sharks interacted with the bait (−4.9%) compared to the other deterrents (−0.3 to −1.9%) (Table S1).

We observed reactions 117 times at an average distance of 1.49 ± 0.05 m (range: 0–6.1 m) from the board and they occurred most frequently with the *Surf*+ (68% of reactions). The *Surf*+ was the only deterrent that demonstrated statistical evidence for changing the percentage of reaction (18%) compared to the control board (2%) (Poisson exact test: *p* = 0.046; *p* > 0.49 for all other deterrents) (Fig. 2B). When the *Surf*+ was active, we observed reactions at an average distance of 1.72 ± 0.05 m (range: 0–6.1 m) from the board (Fig. 2C).

**Figure 3:**
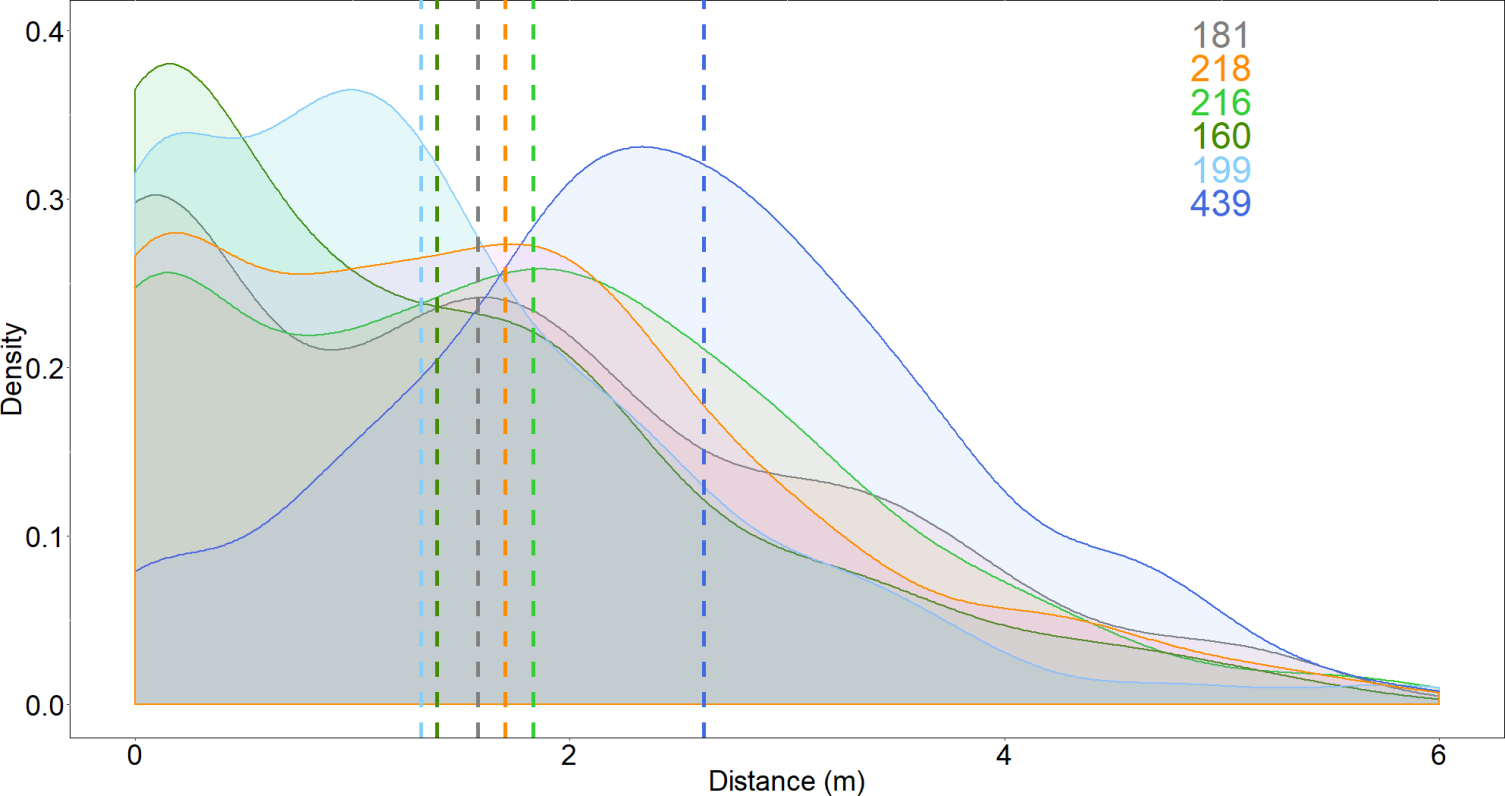
Density distribution of the distance between white shark and the bait. Dash lines represent means for each deterrent. Grey, control; orange, *Chillax Wax*; light green, *magnet band*; green, *magnet leash*; light blue, *Rpela*; blue, *Shark Shield (Ocean Guardian) Freedom*+ *Surf*. Coloured numbers indicate the number of passes from which the density distributions were calculated and match the colours of the deterrents.

**Figure 4:**
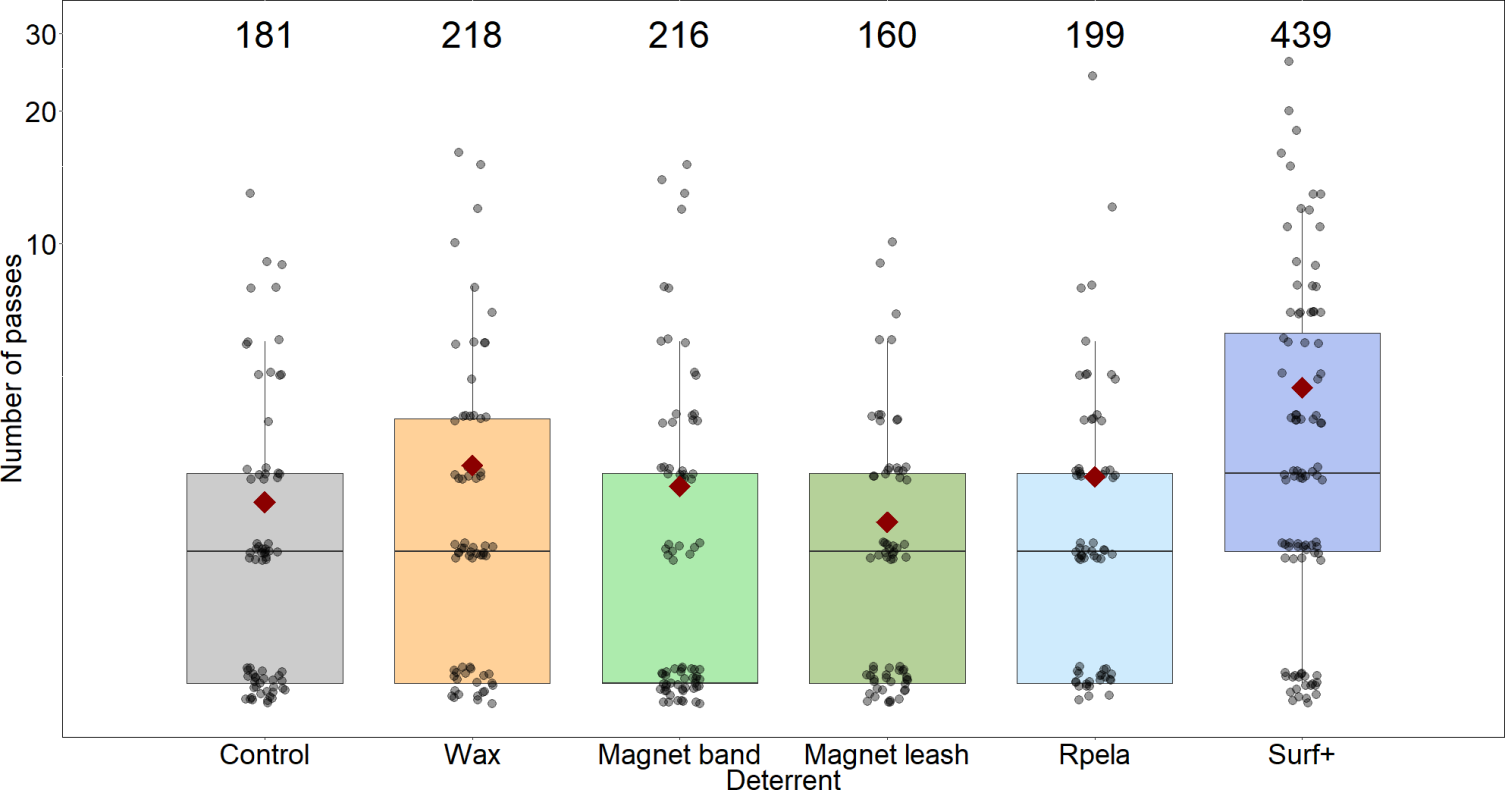
Number of passes trial^−1^ shark^−1^ during 15-minute trials (grey circles; with small ‘jittering’ to improve readability). Median values are indicated by the horizontal bar; length of the box is the inter-quartile range; whiskers represent quartiles; circles are data; and red diamond is the mean. *Y*-axis shown on the log_10_ scale. Grey, control; orange, *Chillax Wax*; light green, *magnet band*; green, *magnet leash*; light blue, *Rpela*; blue, *Shark Shield (Ocean Guardian) Freedom+ Surf*. Numbers indicate total number of passes.

The distance between the shark and the bait, and the number of passes, increased when the *Surf*+ was used compared to the control board, but these variables were not affected by the other deterrents (Figs. 3 and 4). The shark’s distance to the board increased to 2.6 ± 0.1 m when the *Surf*+ was active compared to 1.6 ± 0.1 m with the control board and 1.3 ± 0.1 to 1.8 ± 0.1 m with the other deterrents. The top-ranked model (wAIC_*c*_ = 0.72) included deterrent and explained ∼6.7% of the variance (Table 2B). Again, the *Surf*+ had the largest effect on the number of passes (0.14) compared to the other deterrents (−0.005–0.003) (Table S1).

**Table 2:** Summary of models estimating the effects of deterrents.

**Model**	*k*	AIC_*c*_	Δ**AIC**_*c*_	*w*AIC_*c*_	*R*_*m*_
(a) Probability of the board or bait being touched or bitten by white sharks (binomial error distribution (logit link))					
bait ∼ deterrent	7	178.97	0.00	0.73	23.1
bait ∼ deterrent + trial	8	180.96	1.99	0.27	23.1
bait ∼ 1 (intercept-only)	2	234.48	55.51	<0.01	–
bait ∼ trial	3	236.09	57.12	<0.01	0.6
(b) Distance between white shark and the board (Gaussian error distribution (log link))					
distance ∼ deterrent	8	109.48	0.00	0.72	6.7
distance ∼ deterrent + trial	9	111.42	1.94	0.28	6.7
distance ∼ 1 (intercept-only)	3	164.85	55.37	<0.01	–
distance ∼ trial	4	166.15	56.67	<0.01	0.2
(c) Number of passes by white sharks towards the board (Gaussian error distribution (log link))					
passes ∼ deterrent + trial	9	201.93	0.00	0.53	5.5
passes ∼ deterrent	8	202.69	0.77	0.36	4.4
passes ∼ deterrent + trial + deterrent*trial	14	205.64	3.72	0.08	7.1
passes ∼ trial	4	209.52	7.59	0.01	0.9
passes ∼ 1 (intercept-only)	3	210.05	8.12	0.01	–

**Notes.**

*k* number of model parameters AIC_*c*_ Akaike’s information criterion corrected for small sample size ΔAIC_*c*_ difference in AIC_*c*_ between the current and the top-ranked model *w*AIC_*c*_ model probability *R*_*m*_ marginal (fixed effects) *R*_2_

All models include shark ID as a random effect.

The mean number of passes per trial was highest when the *Surf*+ was active (4.7 ± 0.5), while the number of passes with the other deterrents (2.3 ± 0.3 to 3.1 ± 0.4) was similar to the control board (2.6 ± 0.3). The top-ranked model (wAIC_*c*_ = 0.53) included deterrent and trial, with the deterrent and trial components together explaining ∼5.5% of the variance (Table 2C). However, trial did not strongly affect the number of passes because the next-ranked model did not include trial, had a slightly lower wAIC_*c*_ (0.36), a similar *R*_*m*_, and the trial coefficient was small (0.002). Again, the *Surf*+ had the largest effect on the number of passes (0.176) compared to the other deterrents (−0.04–0.005) (Table S1).

We observed no clear patterns of temporal variation through the trials from the top-ranked models or from plotting the number of passes trial^−1^ or time to interact with the bait (Figs. S2, S3). Only one model had *trial* included in the top-ranked model, but it was not strongly supported compared to the second-ranked model and its goodness of fit was small. Behaviour of the sharks that interacted with the bait in >15 trials showed that the *Surf*+ typically led to more passes and increased distance from the deterrent relative to controls. Neither of these variables consistently increased or decreased across sharks, supporting the lack of temporal effect.

### Power analysis

**Figure 5:**
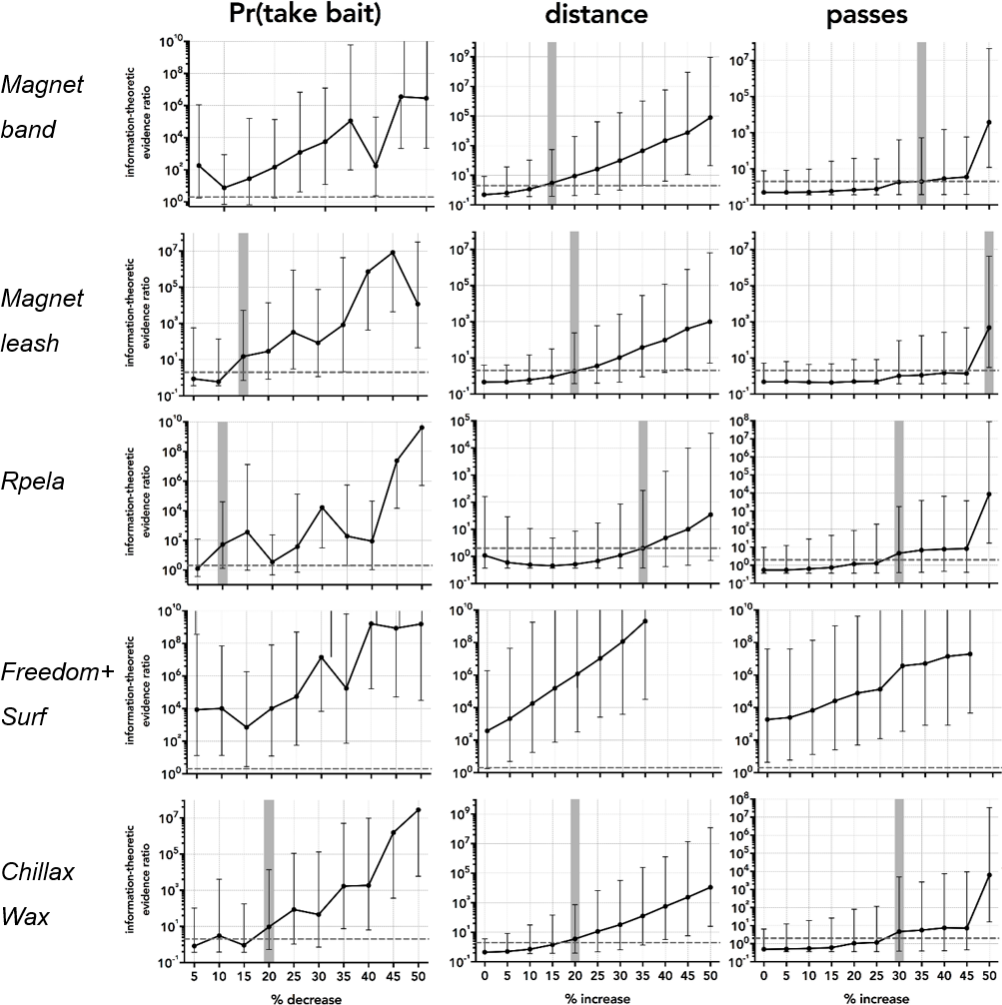
Power analyses results. Information-theoretic evidence ratio of model 3 (response deterrent + shark ID random effect) *versus* the intercept-only model (i.e., *w*AIC_*c*_ of model 3 ÷ *w*AIC_*c*_ of the intercept-only model) for the five deterrent-control pairs and for the four responses of proportion of bait taken (Pr(take bait)), distance to bait, and number of passes, relative to a percentage increase in the effect size relative to the control in increments of 5% from no increase (0) to 50%. The resampled (1,000 times) 95% confidence limits for each percentage increment are indicated by black error bars. Dashed horizontal black line shows the threshold evidence ratio of 2 where model 3 is at least twice as likely to be the true model compared to the intercept-only (i.e., no deterrent effect) model; this is indicated as a vertical horizontal shaded area for each combination and response. Note that we detected a deterrent effect for *Surf*+ (distance and number of passes), so the power analysis indicates an existing effect.

Based on the 297 successful trials (∼50 per deterrent) and the variance structure of these, the *deterrent* effect became detectable when we increased the effect size relative to the controls by between 10 and >50% (Fig. 4). For proportion of bait taken, the smallest detectable effect-size increase was 10% for *Rpela*, and the largest was for *Chillax Wax* at 20% (Fig. 5). For distance to the bait, the smallest detectable effect-size increase was 15% for *magnet band*, and the largest was for *Rpela* at 35% (Fig. 5). Finally, for the number of passes, the increase in effect size required was much larger (30% for *Chillax Wax* and *Rpela*, and 50% for *magnet leash*) (Fig. 5).

## Discussion

Although previous studies have assessed the effectiveness of some personal deterrents, ours is the first to investigate deterrents developed for surfers. We successfully quantified the effectiveness of a combination of deterrents, including new products based on technology thought to affect shark behaviour (*Shark Shield* (Ocean Guardian) *Freedom+ Surf* and *Rpela*), devices that have never been scientifically tested (*SharkBanz bracelet* and *leash*), and novel products (*Chillax Wax*). Our study reveals that while one of the deterrents reduces the probability of white sharks consuming the bait (*Surf*+), the other four deterrents had limited effects on white shark behaviour.

The *Surf*+ had the strongest effect, reducing the percentage of baits taken from 96% to 40%. This increased the number of passes as sharks continued to attempt taking the bait. The other deterrents had limited effects on either the distance to the bait or the number of passes. This suggests that white sharks were not deterred from interacting with the board. Even the *Surf*+, which was the most effective deterrent, did not have a substantial effect on sharks unless they were near, as shown by the short distance from which sharks reacted to this deterrent (∼1.7 m). Although the *Surf*+ affected shark behaviour and reduced the probability of the bait being touched or taken, this deterrent failed to stop sharks in 40% of the trials. Several studies have previously tested the effectiveness of electric deterrents on white sharks and shown variable success (Huveneers et al., 2013b; Kempster et al., 2016; Smit & Peddemors, 2003). The first study assessed *SharkPOD* (a product no longer available) and found that the probability of an attack was reduced from 0.70 to 0.08 when the deterrent was active (88% decrease) (Smit & Peddemors, 2003). More recently, two studies tested the *Freedom7* and highlighted discrepancies in the effectiveness of the device that was related to the bait placement and distance to the electrodes. When the bait was placed next to one electrode, there was an 83% reduction in the proportion of sharks interacting with the bait, concurring with Smit & Peddemors (2003). In Huveneers et al. (2013b), the bait was located ∼2–3 m away from the centre of the electric field produced by the deterrent to reproduce the distance to the head of a user. In this situation and in contrast to Smit & Peddemors (2003) and Kempster et al. (2016), the *Freedom7* did not have a detectable effect on the proportion of times the bait was consumed (Huveneers et al., 2013b). However, a behavioural response was observed with sharks staying farther away when the *Freedom7* was active.

While the waveform and electric field produced by all *Ocean Guardian* products are not different, electrode configuration varies. This results in differences in the maximum field produced and in the location of the electric field in relation to the body of the person using the device and likely explains the contrasting results across previous studies. Combined, Huveneers et al. (2013b), Smit & Peddemors (2003), Kempster et al. (2016), and this study, show that the effective deterrent range of the *Ocean Guardian* waveform depends on how far the bait is from the electrodes, highlighting the importance of carefully considering the position and configuration of the electrodes relative to the object or person intended to be protected by the device. The locations of the *Surf*+ electrodes underneath the surfboard ∼1.2 m apart ensure that surfers are contained by the electric field produced by the deterrent. This electrode configuration is therefore more likely to be suitable than previous products where electrodes were located behind the person wearing the device (e.g., *Freedom7* and *Surf7*—no longer available).

The positions of the *Rpela* electrodes are similar to the *Surf*+; however, the *Rpela* produces an electric field at a higher voltage gradient than the *Surf* + (200 V m^−1^ vs. 115 V m^−1^). Therefore, we expected that the *Rpela* would perform similarly or better than the *Surf*+. However, our experimental observations did not support this and instead we found limited effect when the *Rpela* was active. The *Rpela* was the only other deterrent to which sharks reacted more than the control board (9% vs. 2% of the passes), although this difference was not supported statistically (i.e., we cannot differentiate the two). Mapping the electric field emitted by these two products shows that the *Rpela*’s electric field does not reach as far as the *Surf*+’s despite its stronger voltage gradient. The *Surf*+ had a higher maximum effective distance at 3 V m^−1^ than the *Rpela* (0.7 vs. <0.5 m) (Hart & Ryan, 2018), which might explain the differences in behaviour we observed. The ability to increase the voltage gradient of an electric deterrent is limited because it can cause involuntary muscle spasms of the person wearing it (Bikson, 2004). Kempster et al. (2016) proposed that it could be possible to increase the effectiveness of an electric deterrent by changing the frequency of the electric field discharge. The *Surf*+ discharges at a frequency of ∼1.6 Hz, while *Rpela* has a frequency of ∼14.5 Hz. As a result, the duration of the pulse is also much shorter in the *Rpela* (∼0.2 ms) than the *Surf*+ (∼1.5 ms). Based on the observed difference in effectiveness between the *Rpela* and *Surf*+, the short pulse duration might be less effective at deterring sharks. Alternatively, the longer frequency of the *Surf*+ might be more likely to shock an approaching shark because the animal is able to come closer to the deterrent between pulses, thus feeling the electric pulse more strongly. The pulse duration and frequency are tightly linked, so it is not possible to assess which contributed the most to the discrepancy in shark behaviour between the *Rpela* and *Surf*+. The two deterrents also differ in the type of currents discharged (*Rpela*: direct current; *Surf*+: alternative current), which might have also affected the extent of the sharks’ responses. Whether the lower effectiveness of the *Rpela* is due to the difference in field propagation, pulse type, duration, or frequency is unknown, but the discrepancy between the two products and differences within the *Ocean Guardian* products show the complexity of electric deterrents and the need to ensure that adequate testing is done for all new products before commercial release.

Neither the *SharkBanz bracelet* nor *leash* affected the behaviour of white sharks or reduced the percentage of baits taken. These products rely on permanent magnets (Grade C8 barium ferrite; BaFe_2_O_4_), which have previously been used to overwhelm the electromagnetic sense of sharks. The voltages caused by the induction-based mechanism of the magnets substantially exceed the detection threshold of elasmobranchs (e.g., Jordan, Mandelman & Kajiura, 2011; Kajiura & Fitzgerald, 2009). Permanent magnets have also been shown to elicit avoidance in a range of species, including Galapagos (*C. galapagensis*), hammerhead (*S. mokarran* and *S. lewini*), lemon (*Negaprion brevirostris*), Australian blacktip (*Carcharhinus tilstoni*), grey reef (*C. amblyrhynchos*), bull (*C. leucas*), milk (*Rhizoprionodon acutus*), speartooth (*Glyphis glyphis*), and white sharks (O’Connell et al., 2018; O’Connell et al., 2014a; O’Connell et al., 2014b; O’Connell et al., 2015; O’Connell et al., 2011; O’Connell et al., 2010; Rigg et al., 2009; Robbins, Peddemors & Kennelly, 2011). However, the distance from which sharks reacted to magnets in those studies was small, typically <0.5 m (O’Connell et al., 2014a; Rigg et al., 2009) and the effectiveness of the magnets decreased with increasing shark motivation (Robbins, Peddemors & Kennelly, 2011). Barium-ferrite permanent magnets generate a flux that rapidly decreases in intensity, from ∼1,000 G near the magnet to an amount comparable to the Earth’s magnetic field (0.25–0.65 G) at distances of 0.30–0.50 m (O’Connell et al., 2014a; O’Connell et al., 2014b), showing how rapidly the magnetic field decreases. Sharks would therefore need to be <0.30 m for such magnets to act as real deterrents.

This suggests that magnets are unlikely to be effective at deterring sharks because they will only protect close to the magnet, limiting their applicability as personal deterrents unless stronger magnets can be used or many magnets are positioned on the surfer or board. The latter would add weight to the board and diminish its performance. However, several studies have shown the potential use of strong magnets in combination with visual deterrents to prevent sharks from entering some areas (e.g., beaches, embayments) (O’Connell et al., 2018; O’Connell et al., 2014b). Neodymium-iron-born (‘rare earth’) magnet is the strongest permanent magnet currently available and could be a more powerful deterrent than ferrite magnets. Rare earth magents have much higher surface field strengths than barium-ferrite magnets and might elicit avoidance response from further away (Rigg et al., 2009).

*Chillax Wax* also had limited effect on the behaviour of white sharks and on the likelihood of sharks taking the bait. *Chillax Wax* purports to mask the odour of surfers by overwhelming the shark’s olfactory organs with odour atypical of their natural prey. This combination of eucalyptus, chilli, cloves, cayenne pepper, neem, tea tree oil, citronella, coconut, and beeswax is placed on the deck of surfboards, and the odour is dispersed as surfers paddle or sit on their board. We determined that *Chillax Wax* was not enough to dissuade an approaching shark to take the bait. This might have been caused by the odour of the berley and bait used to run the trials, which could have masked that of the wax; however, berley and bait were necessary to complete sufficient trials. None of the ingredients used in *Chillax Wax* is by itself an established shark repellent and so the product is unlikely to dissuade a shark from biting a surfer. However, *Chillax Wax* might reduce the likelihood of a shark investigating a surfer by masking the latter’s smell. Although sharks could also potentially be attracted to the smell, thus increasing the number of interactions, this did not occur during our trials. More experimental work using *Chillax Wax* is still required to test whether it can reduce the probability of a shark investigating a surfer without relying on bait or berley, although this could be challenging to design and implement.

Although the *Surf*+ reduced the percentage of interactions with the board and bait, it did not completely prevent these interactions. Much of the variation in the models was explained by shark ID (up to three times; results not shown), indicating that behavioural responses were highly variable across individuals. Such individual variation might explain why the *Surf*+ did not stop interactions during all trials. The reason for this variation is unknown and might arise from a combination of different level of satiation, motivation, experiences, dominance hierarchies, or personalities (i.e., behavioural syndrome or consistency of responses across situations). Huveneers et al. (2013b) and other (Huveneers et al., 2013a; Towner et al., 2016) also noted similar intra-specific variability in white sharks, emphasising the need to ensure that shark deterrents are tested on a sufficient number of individuals to identify and account for such individual variability. While the *Surf*+ produced a behavioural reaction in some sharks, it certainly cannot be relied on to prevent shark bites in all situations.

White sharks might have become acclimatised to the deterrent through habituation, or conditioning to the positive rewards from consuming the bait. Such temporal correlation and decrease in the efficacy of an electric field has previously been observed in Galapagos, sandbar (*C. plumbeus*), lemon, and great hammerhead sharks (Brill et al., 2009; O’Connell et al., 2015; Robbins, Peddemors & Kennelly, 2011) and by Kempster et al. (2016) on white sharks. The latter showed that the distance to the deterrent declined with every encounter and suggested that sharks were becoming use to, and more tolerant of, the deterrent. We examined this potential phenomenon, but there was no strong evidence supporting temporal changes in the number of passes and minimal distance. While trial appeared in the top-ranked model for the number of passes, the total model weight (*w*AIC_*c*_) did not strongly support this model over the model not including trial (*w*AIC_c_ difference between models = 0.17) and the percentage of variance explained increased by only 1%. The *trial* coefficient was also small (0.002) and the figure showing the number of passes across trials for the eight sharks interacting with the bait did not show clear trends that suggest any temporal variation. This lack of temporal correlation has previously been observed (Jordan, Mandelman & Kajiura, 2011; Rigg et al., 2009). The small number of food rewards and the alternation of positive and negative reinforcements from the randomly activated deterrents is likely to have prevented habituation.

Although our study did not show that the *magnet band* and *leash*, *Chillax Wax*, and *Rpela* had any observable effects on the response of white sharks, it is possible that these deterrents have small effects that we could not detect with the 50 trials. The estimated minimum effect size across responses shows that our study design would not have been able to detect a difference of a <30% increase in the number of passes and a 15–35% increase in the approach distance between the shark and the bait. This does not infer that additional trials would have necessarily resulted in any effects being detected, but that more than 50 trials would be required to detect changes of a magnitude <15%, if present. Although the number of trials in our design was insufficient to detect effects <15 %, the public likely expects shark deterrents to reduce the probability of being attacked or bitten by >15%, which our study was able to detect. However, if other agencies require testing for smaller effect sizes, greater samples sizes than what we tested would be required. Behavioural studies on large predatory sharks like white sharks can be logistically challenging and expensive because it can be difficult to do a large number of trials given the inherently low abundance of sharks and their unpredicatibility in interacting with experimental equipment. Power analyses like ours will enable future studies to consider the number of trials required to assess the efficacy of shark deterrents.

Since shark location during our experiments was unknown, we could not account for possible interactions between sharks. However, while we observed several sharks within 20 m of the equipment, it was rare that more than one shark approached the bait simultaneously. Our results could also have been potentially influenced by the study site being where cage-diving usually occurs and our reliance on berley to attract sharks. The need for sufficient replicates required choosing a place where many sharks aggregate and using berley to attract them. We also acknowledge that testing at a fur seal colony using natural prey as an attractant in an area where white sharks feed presents an extreme situation, which is a different context to that of most swimmers or surfers, and that behavioural response of sharks might depend on context (Huveneers et al., 2013b). In the case of the *Surf*+, its ability to reduce the percentage of bait taken might improve when sharks are less motivated, whereas the other deterrents could begin to demonstrate effects. Although future research is necessary to provide more insight into why white sharks seize humans, white shark interactions with humans are highly variable. They can range from situations where a shark does not closely approach a person, to targeted strikes potentially motivated by hunger. Therefore, the context in which deterrents are tested should not be directly extrapolated to all shark-bite situations and our results should be presented in this context.

## Conclusion

The rise in the number of shark bites worldwide and in Australia has led to global development of shark-mitigation measures and the commercial availability of several personal deterrents. Our study clearly shows that while some products are capable of affecting the behaviour of sharks and can reduce the risk of a shark bite, others did not have the advertised outcome. Manufacturers should consider these results to assess the suitability of their products and gauge whether changes are required to ensure their intended performance. Our results will allow private and government agencies to make informed decisions about the use of these devices for occupational activities and enable the public to make appropriate decisions about the use and suitability of these five products.

##  Supplemental Information

10.7717/peerj.5554/supp-1 Supplemental Information 1 Supplementary File

## Additional Information and Declarations

### Field Study Permissions

The following information was supplied relating to field study approvals (i.e., approving body and any reference numbers): Field experiments were approved by the Department of Environment, Water and Natural Resources permit M26609.

### Data Availability

The following information was supplied regarding data availability: GitHub: https://github.com/cjabradshaw/whitesharkdeterrents.
