# Supplementary material for: Effectiveness of five personal shark-bite deterrents for surfers
Source: PeerJ. 2018 Aug 31;6:e5554. doi: 10.7717/peerj.5554 (PMC6120439; doi:10.7717/peerj.5554)
Supplement: Supplemental Information 1 [file peerj-06-5554-s001.docx]

Figure S1. Number of passes of white sharks (*Carcharodon carcharias*) during the 297 trials according to approach type and level of intent (white = low; grey = medium; black = high).


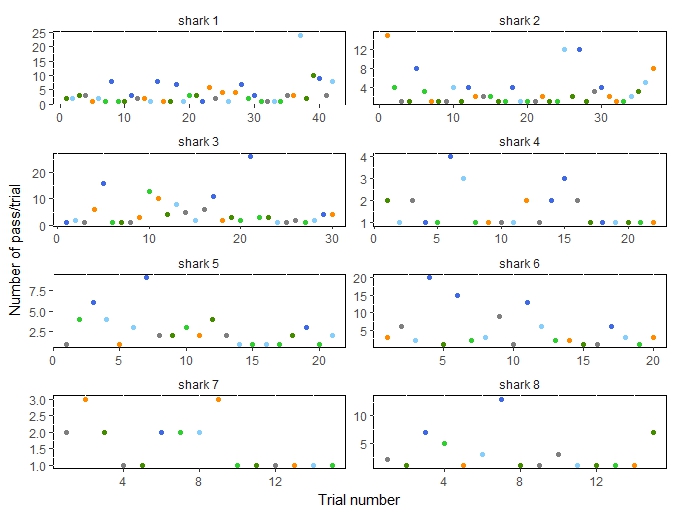


Figure S2. Number of passes during the 15-minute trials for the eight white sharks that interacted with the boards on 15 trials or more. Grey = control; orange = *Chillax Wax*; light green = *magnet band*; green = *magnet leash*; light blue = *Rpela*; blue = *Shark Shield* (*Ocean Guardian*) *Freedom+ Surf*.


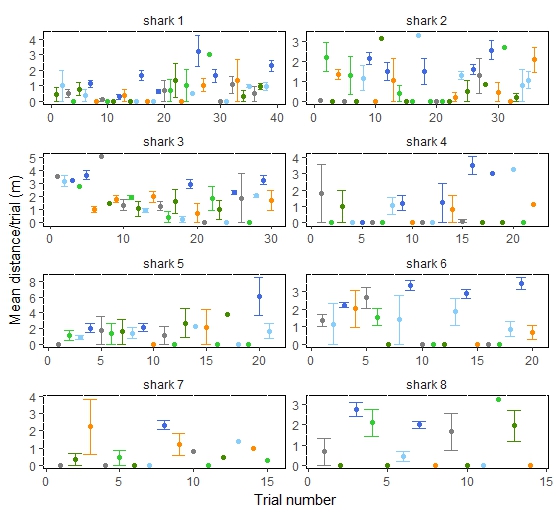


Figure S3. Mean distance between white shark and the bait for the eight sharks that interacted with the boards on 15 trials or more. Error bars represent standard errors. Grey = control; orange = *Chillax Wax*; light green = *magnet band*; green = *magnet leash*; light blue = *Rpela*; blue = *Shark Shield* (*Ocean Guardian*) *Freedom+ Surf*.

Table S1. Estimated deterrent level coefficients (*β*) and their standard errors (SE), *z*-values of factors included in the top-ranked model (indicated for each variable), and the individual coefficient Type I error estimate (*P*) when relevant.

| **Level** | ***β*** | **SE** | ***z*** | ***P*** |
| --- | --- | --- | --- | --- |
| *Bait ~ deterrent* |  |  |  |  |
| intercept | 3.061 | 1.013 | 3.02 | 0.003 |
| *Chillax Wax* | -0.305 | 1.065 | -0.29 | 0.775 |
| *Magnet band* | -1.855 | 1.036 | -1.79 | 0.073 |
| *Magnet leash* | 0.628 | 1.236 | 0.51 | 0.612 |
| *Rpela* | -1.337 | 1.047 | -1.28 | 0.202 |
| *Surf+* | -4.906 | 1.088 | -4.51 | < 0.001 |
|  | | | | |
| *Distance ~ deterrent* | | | | |
| intercept | 3.181 | 0.032 | 98.34 |  |
| *Chillax Wax* | -0.005 | 0.036 | -0.14 |  |
| *Magnet band* | 0.003 | 0.034 | 0.09 |  |
| *Magnet leash* | -0.014 | 0.039 | -0.37 |  |
| *Rpela* | -0.046 | 0.034 | -1.38 |  |
| *Surf+* | 0.140 | 0.031 | 4.54 |  |
|  | | | | |
| *Passes ~ deterrent + trial* | | | | |
| intercept | -0.408 | 0.056 | -7.35 |  |
| *Chillax Wax* | 0.050 | 0.057 | 0.88 |  |
| *Magnet band* | 0.009 | 0.056 | 0.15 |  |
| *Magnet leash* | -0.034 | 0.057 | -0.60 |  |
| *Rpela* | 0.032 | 0.058 | 0.55 |  |
| *Surf+* | 0.176 | 0.056 | 3.16 |  |
| *Trial* | 0.002 | 0.001 | 1.64 |  |
